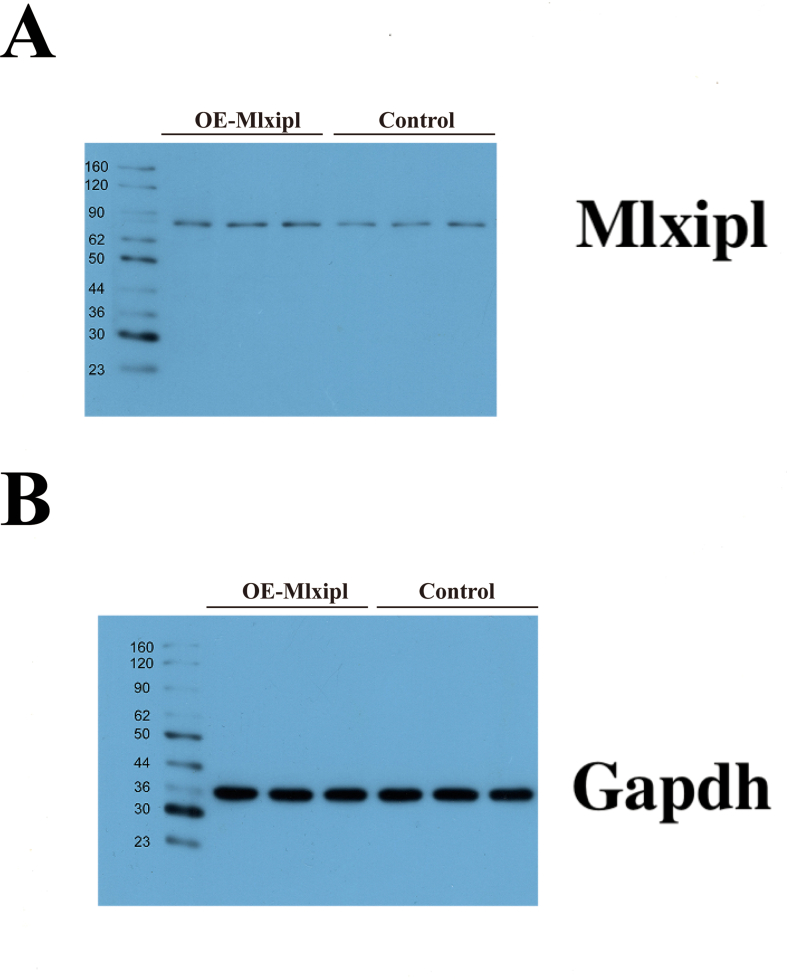# Sox11 deficiency induces paravertebral muscle injury and scoliosis via Mlxipl upregulation

**DOI:** 10.1016/j.gendis.2025.101970

**Published:** 2025-12-12

**Authors:** Ruohao Wu, Wenting Tang, Yu Li, Zihao Deng, Jing Zhang, Xiaojuan Li, Chunwei Cao, Liyang Liang

**Affiliations:** a Department of Children's Medical Center, Sun Yat-Sen Memorial Hospital, Sun Yat-Sen University, Guangzhou, Guangdong 510120, China; b Department of Research and Molecular Diagnostics, Sun Yat-Sen University Cancer Center, Sun Yat-Sen University, Guangzhou, Guangdong 510060, China; c Sino-French Hoffmann Institute, School of Basic Medical Science, Guangzhou Medical University, Guangzhou, Guangdong 511436, China; d Guangdong Provincial Key Laboratory of Malignant Tumor Epigenetics and Gene Regulation, Guangdong-Hong Kong Joint Laboratory for RNA Medicine, Medical Research Center, Sun Yat-Sen Memorial Hospital, Sun Yat-Sen University, Guangzhou, Guangdong 510120, China; e Department of Cellular and Molecular Diagnostics, Sun Yat-sen Memorial Hospital, Sun Yat-sen University, Guangzhou, Guangdong 510120, China; f Guangzhou National Laboratory, Guangzhou, Guangdong 510320, China

Proteins encoded by *SRY-related Box* (*SOX*) genes have emerged as a pivotal family of transcription factors, orchestrating diverse processes critical to human organ development. Abnormalities in these *SOX* genes are linked to varieties of rare disorders, termed SOXopathies. Musculoskeletal malformation is a featured phenotypes shared by those SOXopathies; among those musculoskeletal malformations, scoliosis is one of the high-incidence phenotypes in patients with SOXopathies.

Previous studies have established that disruptions in SOX genes, leading to impaired development of vertebral cartilage, underlie scoliosis in SOXopathies.^1^ Notably, clinical evidence, including the heterozygous SOX11 deletion reported by Hempel et al^2^ and our patient, a child with a pathogenic SOX11 variant (p.Y116C) who presented with scoliosis (Fig. 1A), further supports this link. Meanwhile, accumulating data indicate that pathological changes in the paravertebral muscles (PVMs) also contribute critically to scoliosis pathogenesis.^3^ Yet, the role of PVM abnormalities in SOXopathies or individuals with SOX11 deficiency remains largely unexplored. Here, we generated a heterozygous Sox11-deficient (*Sox11*^*+/−*^) mouse model that recapitulates the scoliosis phenotype. We discovered that Sox11 deficiency-induced PVM injury significantly contributes to the etiopathogenesis of spinal deformity, yielding important insights into different etiologies and pathogenesis in the spectrum of SOXopathies-related scoliosis.

**Figure 1:**
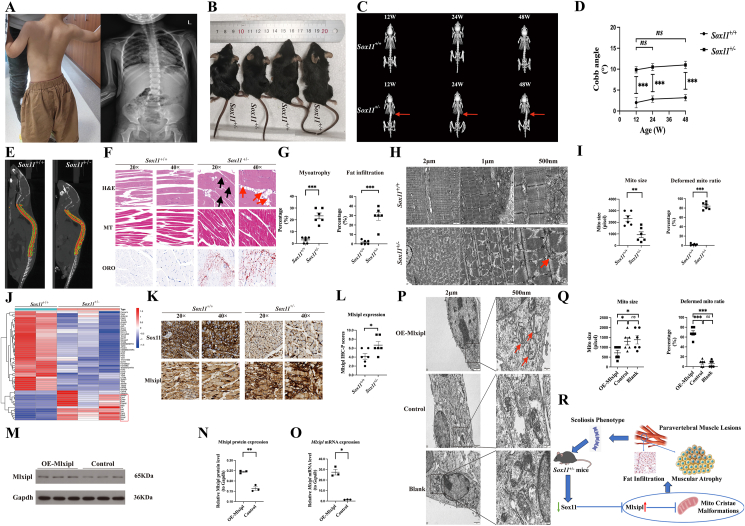
The biological effects and mechanisms of Sox11 heterozygous deficiency for the pathogenesis of SOXopathies-related scoliosis by the overexpression of Mlxipl in PVM. **(A)** A clinical patient who harbored a pathogenic missense *SOX11* variant (NM_003108.4: c.347A > G, p.Y116C) shows short stature and scoliosis phenotype (*left*) that further confirmed by X-ray tests (*right*). **(B)** The appearances of *Sox11*^+/+^ and *Sox11*^+/−^ mice at 12 weeks. **(C)** Representative dorsal micro-CT images of *Sox11*^+/+^ and *Sox11*^+/−^ mice at 12, 24 and 48 week-of-age, respectively. Red arrows indicate scoliosis. **(D)** Longitudinal observations at 12, 24 and 48 weeks showing cobb angle measurements between *Sox11*^+/+^ and *Sox11*^+/−^ mice. **(E)** The muscle volumes of PVM were measured by calculating green area and compared between *Sox11*^+/+^ and *Sox11*^+/−^ mice. **(F)** H&E, MT and ORO stained PVM sections of *Sox11*^+/+^ and *Sox11*^+/−^ mice. Black arrows showing myofiber atrophy. Red arrows showing fat infiltration. **(G)** Quantity assessment of percentages of myofiber atrophy (3.00% ± 1.00% *vs.* 23.33% ± 2.47%, *P* < 0.001) and fat infiltration (1.83% ± 0.87% *vs.* 29.17% ± 4.17%, *P* < 0.001) in PVM samples of *Sox11*^+/+^ and *Sox11*^+/−^ mice. **(H)** TEM of muscle cells in PVM slices of *Sox11*^+/+^ and *Sox11*^+/−^ mice demonstrating mitochondrial morphology. Red arrows revealing a small-size mitochondrion without normal cristae structures in PVM muscle cells of *Sox11*^+/−^ mice. **(I)** Analysis of the size (area) of single mitochondrion (933.33 ± 248.55 pixel *vs.* 2333.33 ± 238.98 pixel, *P* < 0.01) and the percentage of deformed-cristae mitochondrion (84.17% ± 3.08% *vs.* 1.83% ± 0.79%, *P* < 0.001) in PVM muscle cells of *Sox11*^+/+^ and *Sox11*^+/−^ mice using Image J. **(J)** All differentially expressed genes (|log_2_FC| >1, *P* < 0.05; down-regulation: 40 genes and up-regulation: 15 genes) in developmental PVM samples of littermate *Sox11*^+/+^ and *Sox11*^+/−^ mice were shown in heatmap. Red box indicating all 15 up-regulation genes in PVM samples of littermate *Sox11*^+/+^ and *Sox11*^+/−^ mice identified by RNA-seq analysis. **(K)** Representative IHC images showing the expression levels of Sox11 and Mlxipl in developmental PVM sections of *Sox11*^+/+^ and *Sox11*^+/−^ mice. **(L)** Quantity evaluation of Mlxipl IHC-P value (6.67 ± 0.80 *vs.* 4.17 ± 0.65, *P* < 0.05) in PVM slices of *Sox11*^+/+^ and *Sox11*^+/−^ mice. **(M,N)** WB analysis (0.24 ± 0.004 *vs.* 0.16 ± 0.009, *P* < 0.01) and **(O)** qRT-PCR (27.44 ± 2.66 *vs.* 1.40 ± 0.10, *P* = 0.01) showing Mlxipl overexpression in transfected OE-Mlxipl cell group. **(P)** TEM of mouse myogenous cells in OE-Mlxipl, Control, and Blank groups revealing mitochondrial morphology. Red arrows revealing a relatively small-size mitochondrion with abnormal cristae structures. **(Q)** Analysis of the size of single mitochondrion (716.67 ± 130.17 pixel *vs.* 1300.00 ± 182.57 pixel, *P* < 0.05; 716.67 ± 130.17 pixel *vs.* 1383.33 ± 216.67 pixel, *P* < 0.05) and the percentage of deformed-cristae mitochondrion (68.34% ± 4.53% *vs.* 7.78% ± 2.68%, *P* < 0.001; 68.34% ± 4.53% *vs.* 5.83% ± 2.71%, *P* < 0.001) in OE-Mlxipl and Control/Blank groups using Image J. **(R)** A schematic illustrates how Sox11 deficiency induced PVM injury via up-regulating Mlxipl, promoting the development of scoliosis underlying SOXopathies conditions. ∗, *P* < 0.05; ∗∗, *P* < 0.01; ∗∗∗, *P* < 0.001; *ns*, *P* > 0.05. **Abbreviations:** PVM, paravertebral muscle; micro-CT, microfauna computed tomography; hematoxylin-eosin; MT, masson's trichrome; ORO, oil-red-o; TEM, transmission electron microscopy; Mito, mitochondrion; IHC, immunohistochemistry; IHC-P, immunohistochemistry protein expression; qRT-PCR, quantitative real-time polymerase chain reaction; WB, western blotting.

*Sox11*^*+/−*^ and wild-type (*Sox11*^*+/+*^) offspring were generated by intercrossing the original transgenic *Sox11*^*+/−*^ mice. By 12 weeks of age, those *Sox11*^+/−^ mice displayed body twisting along with a notably shorter body length compared with *Sox11*^+/+^ (Fig. 1B; Fig. S1A). From the side view, we observed a thoracolumbar scoliosis phenotype existed in the *Sox11*^+/−^ mice (Fig. S1B). Using microfauna computed tomography (micro-CT), and based on the established assessment criterion for scoliosis severity (cobb angle: mild, <10°; moderate, 10°–20°; severe, 20°–40°; profound, >40° ), we confirmed that *Sox11*^+/−^ mice exhibited mild-moderate scoliosis (cobb angle: 9° to 12°) since 12-week-old (Fig. 1C and D; Fig. S1C). The onset age of scoliosis identified in those *Sox11*^+/−^ mice were comparable to that human individuals with SOX11 syndrome and scoliosis mentioned above. The observed resemblance underscores the potential of these animal models in uncovering the natural progression and molecular underpinnings of scoliosis related to human SOX11 syndrome. Notably, quantitative analysis of spinal parameters, including spine length, volume and bone mineral density, revealed no significant differences between *Sox11*^+/−^ and *Sox11*^+/+^ mice (Fig. S2A and B), indicating the development of scoliosis in *Sox11*^+/−^ mice might be not associated with structural abnormalities or impaired bone mineralization of the spine. We next investigated whether *Sox11*^*+/−*^ mice exhibited pathological changes in PVM. Sagittal micro-CT analysis revealed that the paravertebral muscle (PVM) volume at the thoracolumbar region was approximately 12.3% smaller in *Sox11*^*+/−*^ mice than in *Sox11*^*+/+*^ controls (162.95 ± 4.67 mm^3^ *vs.* 185.86 ± 4.39 mm^3^; *P* = 0.005), representing a significant reduction of about 23 mm^3^ (Fig. 1E; Fig. S2C). These findings suggest a potential association between the PVM injury and the development of scoliosis in *Sox11*^+/−^ mice.

To further determine the histopathological and cellular alterations underlying the reduced muscle volume of PVM in *Sox11*^+/−^ mice with scoliosis, we performed hematoxylin-eosin, masson's trichrome and oil-red-o staining on PVM tissues from *Sox11*^*+/−*^ mice, and the results showed extensive percentages of myofiber atrophy (3.00% ± 1.00% *vs*. 23.33% ± 2.47%, *P* < 0.001) with extensive fat infiltration (1.83% ± 0.87% *vs*. 29.17% ± 4.17%, *P* < 0.001) in the PVMs of *Sox11*^*+/−*^ mice (Fig. 1F and G). Since the increased myofiber atrophy concurrent with an imbalance in fat accumulation, we postulated the existence of an underlying metabolic perturbation at the cellular level. We thus investigate the mitochondrial morphology in the PVM cells of *Sox11*^*+/−*^ mice by using transmission electron microscopy (TEM). *Sox11*^*+/−*^ mice exhibited widespread myofibrillar disorganization with disrupted gap junctions, consistent with the histopathological findings of myofiber atrophy. Importantly, the size of the mitochondria in PVM were markedly reduced in *Sox11*^*+/−*^ mice (933.33 ± 248.55pixel *vs*. 2333.33 ± 238.98pixel, *P* = 0.002). Notably, a large proportion of mitochondria in *Sox11*^*+/−*^ mice failed to develop normal cristae structures (84.17% ± 3.08% *vs*. 1.833% ± 0.79%, *P* < 0.001) (Fig. 1H and I), indicating that mitochondrial malformations, particularly defects in cristae structure, may impair energy metabolism and lead to the pathological changes observed in the PVM of *Sox11*^*+/−*^ mice, including myofiber atrophy and fat accumulation.

To further investigate the underlying molecular mechanisms associated with mitochondrial cristae malformations and related metabolic dysregulation in the PVM of *Sox11*^+/−^ mice, RNA-seq was performed to identify differentially expressed genes (DEGs) between *Sox11*^*+/−*^ and *Sox11*^*+/+*^ mice, as well as to screen for enriched gene ontology (GO) biological terms associated with the DEGs. A total of 40 down-regulated DEGs and 15 up-regulated DEGs were identified (Fig. 1J), revealing the down-regulated DEGs were predominantly enriched in pathways related to organ morphogenesis, whereas the up-regulated DEGs were mainly associated with lipid metabolic process (Fig. S3A and B). Subsequently, a comprehensive literature review analysis was conducted on the 15 up-regulated DEGs, revealing that five genes (*Car4*/*C3*/*Car3*/*Klhdc7a*/*Xist*) were not associated with lipid metabolism regulation or mitochondrial biogenesis, nine genes (*G0s2*/*Gpd1*/*Pnpla2*/*Adhfe1/Sctr*/*Cidec*/*Adig*/*Irs4*/*Ces1d*) were solely linked to lipid regulation, and one gene, *Mlxipl*, was implicated in both lipid regulation and mitochondrial biogenesis, with a specific role in cristae formation ^4^^,^^5^ (Fig. S3C). Thus, Mlxipl is a strong candidate responsible for the cristae abnormalities observed in the PVM of *Sox11*^*+/−*^ mice. To validate the RNA-seq findings, immunohistochemistry (IHC) experiments were then conducted on PVM samples. IHC showed that Sox11 expression was significantly lower in *Sox11*^*+/−*^ mice (2.33 ± 0.33 *vs*. 7.50 ± 0.67, *P* < 0.001), while Mlxipl expression was significantly higher (6.67 ± 0.80 *vs*. 4.17 ± 0.65, *P* = 0.036) (Fig. 1K and L; Fig. S3D). These results confirm the differential expression patterns observed in the RNA-seq data.

Moreover, to investigate the functional impact of Mlxipl overexpression on mitochondrial cristae formation in mouse myogenesis cells, the coding sequence of mouse *Mlxipl* gene was cloned into the SBI-piggyBac plasmid vector and stably transfected into C2C12-derived myogenic cells to induce stable Mlxipl overexpression. Analyses confirmed that Mlxipl expression was significantly upregulated in C2C12 myoblasts transfected with the Mlxipl overexpression plasmid (OE-Mlxipl group) compared to those transfected with empty vector (Control group), both at the protein (Fig. 1M and N; Fig. S4A and B) and mRNA levels (Fig. 1O). These results indicate successful establishment of a stable Mlxipl-overexpressing muscle cell-model *in-vitro*. Using TEM, we observed that OE-Mlxipl group exhibited a higher proportion of small-size mitochondria with impaired cristae formation compared to both the Control and the PBS-treated blank groups (Fig. 1P, Q). These findings were consistent with the mitochondrial abnormalities observed in the PVM of *Sox11*^+/−^ mice. While this consistency strengthens the relevance of our model, it is important to note a key limitation inherent to our approach: the absence of direct evidence from human PVM samples. Although the scoliosis phenotype in a patient with a SOX11 missense variant provides clinical association, we cannot yet confirm that the same molecular alterations occur in human PVM. Nevertheless, future studies on human tissue samples are essential to fully bridge this translational gap. Meanwhile, C2C12 cell is a myoblast cell-line that differs from mature muscle cells, which warrants experiments with mature muscle cells to further confirm our *in-vitro* observations.

In conclusion, our study establishes the role of PVM injury and Mlxipl dysregulation in the pathogenesis of SOX11 syndrome-related scoliosis (Fig. 1R), thereby bridging a critical gap in understanding SOXopathy manifestations. Importantly, given that metformin is known to ameliorate metabolic disturbances by targeting Mlxipl, our findings linking Mlxipl overexpression to scoliosis suggest a promising translational avenue. This opens up the possibility of repurposing anti-metabolic therapies, such as metformin, for the management of SOX11 syndrome-related scoliosis.

## CRediT authorship contribution statement

**Ruohao Wu:** Writing – original draft, Methodology, Funding acquisition, Formal analysis, Data curation. **Wenting Tang:** Project administration, Investigation. **Yu Li:** Project administration, Investigation. **Zihao Deng:** Visualization, Software, Formal analysis. **Jing Zhang:** Formal analysis. **Xiaojuan Li:** Writing – review & editing, Resources, Funding acquisition. **Chunwei Cao:** Writing – review & editing, Supervision, Resources. **Liyang Liang:** Writing – review & editing, Visualization, Validation, Supervision, Resources, Funding acquisition, Conceptualization.

## Ethics statement

All procedures of this study involving in human subjects were done in agreement with the Declaration of Helsinki and approved by the Ethics Committee of Sun Yat-sen Memorial Hospital, Guangzhou, China (Approval Number: SYSKY-2023-709-01). All mouse procedures in this research were reviewed and approved by the Animal Care and Use Committee of the RUIYE Animal Laboratory, Guangzhou, China (Approval Number: RYEth-20230207154).

## Data availability

The original contributions presented in the study are included in the article and its supplementary materials.

## Funding

This work was supported by the grants from the Medical Scientific Research Foundation of Guangdong Province, China (Grant No. B2025526) and the National Natural Science Foundation of China (Grant No. 81800155 and 32170612).

## Conflict of interests

The authors declare that they have no conflict of interests.

## Figures and Tables

**figs1:**
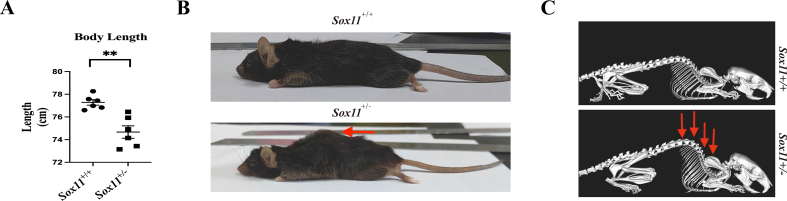


**figs2:**
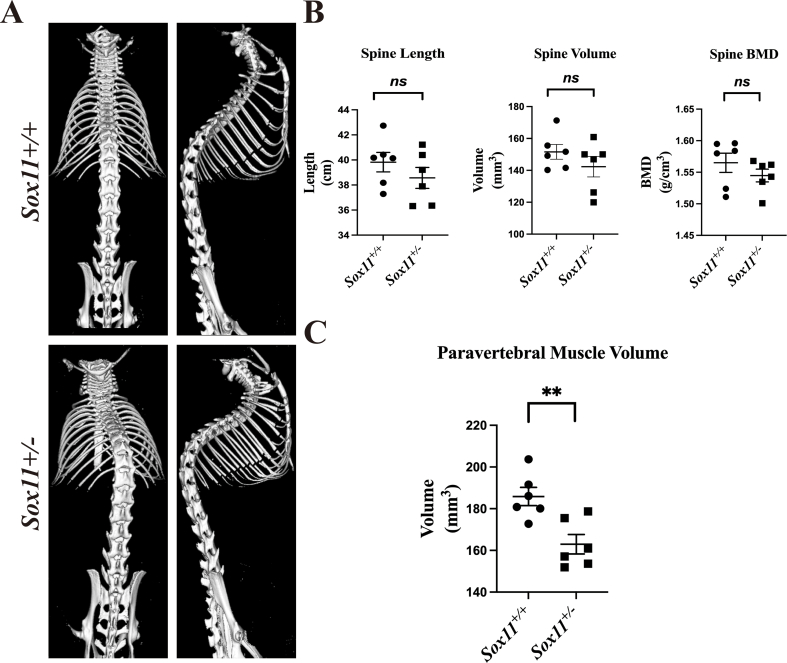


**figs3:**
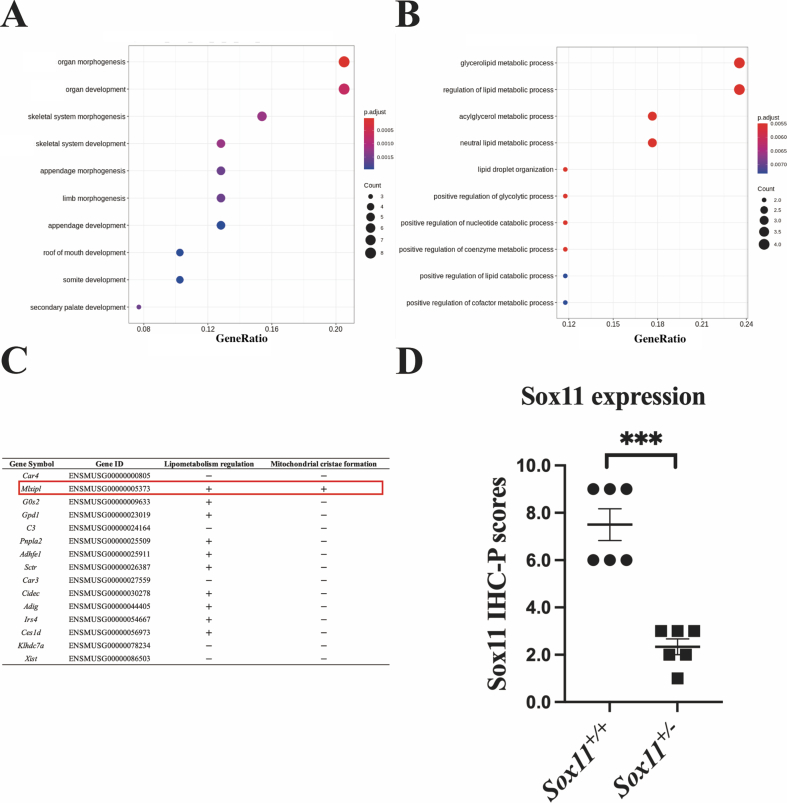


**figs4:**