# Supplementary material for: Sox11 deficiency induces paravertebral muscle injury and scoliosis via Mlxipl upregulation
Source: Genes Dis. 2025 Dec 12;13(3):101970. doi: 10.1016/j.gendis.2025.101970 (PMC12859181; doi:10.1016/j.gendis.2025.101970)
Supplement: Multimedia component 1 [file mmc1.docx]

**Materials and methods**

**Generation and care of mice**

Original Sox11 heterozygous knockout (Sox11^+/-^) mice were generated by Professor Chunwei Cao using CRISPR-Cas9 gene-editing technology. The mice were housed in a specific-pathogen-free (SPF) facility under controlled temperature and humidity conditions, with a 12-hour light/dark cycle. Heterozygous and wild-type (Sox11^+/+^) offspring were obtained by breeding Sox11^+/-^ male and female mice.

**Mouse genotyping**

Genomic DNAs were obtained from tail tips of mice for genotyping identification. The polymerase chain reaction (PCR) conditions for genotyping included an initial 10 min denaturation step at 95 ℃, followed by 35 cycles of 30 s at 94 ℃, 40 s at 58 ℃, and 60 s at 72 ℃ with the final 5 min extension step at 72 ℃. The PCR 20 μL reaction mixtures mainly contained 5% dimethyl sulfoxide, standard puffer, and a primer with 0.25 μM concentration. The primers used for genotyping were presented in **Supporting File 1**. A representative image of gel electrophoresis revealed that a 553-bp fragment was indicative of the targeted *Sox11^+/-^* allele; while, a single 390-bp fragment was indicative of the *Sox1^1+/+^* allele (**Supporting File 2**).

**Microfauna computed tomography (micro-CT) analysis**

Radiographs of the skeleton system and related tissues of *Sox11^+/-^* and *Sox1^1+/+^* mice were generated by using a Quantum GX2 Micro-CT system (PerkinElmer, Waltham, USA) with auto exposure parameters (Voltage: 90 kV, Current: 88 μA, FOV: 36 mm, San Mode: Standard 2 min, Stitching Scan: 5). For avoiding stiffness of the skeleton, all radiographs were taken immediately after the subject mice were euthanized with isoflurance provided by a rodent gas anesthesia system (Revvity, Shanghai, China). Spinal parameters, including Cobb angle, spine length, spine volume, spine bone mineral density (BMD) and muscle volume of paravertebral muscle (PVM), were all measured on high resolution X-ray and 3D reconstruction images with the analysis platform of Quantum GX2 Micro-CT system (PerkinElmer, Waltham, USA).

**Histological staining and histopathological analysis**

After harvest, fixed PVM muscle samples were embedded with paraffin and cut into PVM slices with 5 μm thickness. PVM slices were firstly stained with hematoxylin-eosin (H&E) as previously described[1]. Then, the extent of myofiber atrophy and the accumulation of lipid droplets (fat infiltration) in PVMs were further determined by masson’s trichrome (MT) and oil-red-o (ORO) staining, respectively. Briefly, for ORO staining, we firstly mixed the ORO stock solution (G-clone, Beijing, China) with double distilled water as the ratio of 3:2 to make ORO working solution. Air-dried and deparaffinized PVM slices were rinsed with double distilled water and 60% isopropanol, respectively. Then, rinsed slices were stained with ORO working solution for 15 min. After differentiation by 60% isopropanol and rinsing by double distilled water, the PVM slices were stained with hematoxylin solution for 30 s and washed by tap-water. Finally, the slices were mounted by neutral resin. For MT staining, deparaffinized PVM slices were rinsed with double distilled water and stained with Weigert’s iron hematoxylin solution for 10 min. Then, washed slices were stained in Biebrich scarlet-acid fuchsin solution for 10 min and differentiated in phosphomolybdic-phosphotungstic acid solution for 15 min. After staining with aniline blue solution for 5 min, the washed PVM slices were dehydrated through 95% alcohol and absolute alcohol very quickly, and rinsed by xylene. Finally, the slices were mounted with resinous mounting medium. For histopathological analysis, images of 5-6 different field with 10×, 20× and 40× magnifications per sample were taken under an optical microscope equipped with a digital image capture system (Leica Camera, Wetzlar, Germany). Then, semi-quantitative analyses of PVM histology were conducted by two independent experienced pathologists with a blinded fashion using Image J software (<http://imagej.nih.gov/ij/>). In short, a semi-quantitative scoring system for PVM histology (including H&E, MT and ORO staining) was employed to assess the extent of key pathological features. The extent for each feature (myofiber atrophy or fat infiltration) was assigned based on the estimated percentage of the total cross-sectional area (CSA) affected and made as follows: **Normal**, absence of the pathological feature or involvement of less than 5% of the muscle fibers. **Mild**, the pathological feature affects approximately 5% to 10% of the muscle fibers within the section. **Moderate**, the pathological feature involves approximately 10% to 20% of the muscle fibers. **Extensive**, the pathological feature is presented in more than 20% of the muscle fibers.

**Transmission electron microscope (TEM) and cellular pathological analysis**

To perform TEM analysis, a block of PVM was immediately removed from paraspinal area, and grinded into rice-shaped tissue particles in 4% paraformaldehyde fixative with 1% osmic acid and PBS at 4 ℃. Then, grinded PVM tissues were dehydrated with the ethanol (gradient from 50% to 100%) and embedded by epon resin. Finally, sliced ultra-thin PVM sections were stained with lead citrate and 2% uranyl acetate. For cellular pathological analysis, images with different magnifications (2 μm, 1 μm and 500 nm) per sample were taken under an TEM equipped with a digital image capture system (ThermoFisher Scientific, Waltham, USA). Then, semi-quantitative analyses of PVM cellular changes were conducted by two independent experienced researchers with a blinded fashion using Image J software. In short, a quantitative analysis of mitochondrial size and morphology was performed on digitally captured micrographs at a standard magnification (2 μm, 1 μm or 500 nm) using Image J software. For each sample, a minimum of 50 mitochondria from 10-15 randomly selected fields of view were analyzed by two independent observers who were blinded to the experimental groups. For mitochondrial size, the CSA of individual mitochondria was measured in pixels. Only mitochondria with a clearly defined, intact outer membrane and a complete cross-section within the micrograph were included to avoid measurement bias from obliquely sectioned organelles. For proportion of deformed mitochondria that failed to develop normal cristae structures, we used the percentage of total numbers of abnormal mitochondria per sample to assess it.

**RNA-seq and related bioinformatic analysis**

For RNA-seq analysis, total cellular RNA from PVM tissues of littermate *Sox11^+/+^* and *Sox11^+/-^* mice was first isolated with TRIzol reagent (Ambion, Austin, USA) following the manufacturer’s protocol. The concentration and integrity of isolated RNA were determined using the Agilent Bioanalyzer 2100 system (Agilent, Santa Clara, USA). Then, qualified RNA samples (RIN ≥ 7) were used for construction of sequencing library via Illumina mRNA-seq Lib Prep Kit (Illumina, San Diego, USA) with BGI platform adapters (BGI, Shenzhen, China). The process of sequencing was conducted on the DNBSEQ-T7 platform (BGI, Shenzhen, China), and the sequencing data were processed as follows: sequencing base quality analysis and filtering using Trim Galore tool (version: 0.61, http://www.bioinformatics.babraham.ac.uk/projects/trim_galore) RNA-seq reads were aligned to the GRCm38 (mm10) reference genome using STAR aligner (v2.7.10) [2], with subsequent transcript quantification achieved through gene-level read enumeration via featureCounts (Subread package v2.0.3)[3]. The DESeq2 package (v1.32) was then employed for differential expression analysis, and differentially expressed genes (DEGs) were identified based on thresholds of *P* < 0.05 and |log₂FC| > 1[4]. The DEGs were visualized via heatmaps using DESeq2 package (version: 1.32)[4]. Gene ontology (GO) functional enrichment analysis were performed and visualized via bubble plots using clusterProfiler (version: 3.10)[5].

**Immunohistochemistry (IHC) analysis**

To further validate the RNA-seq findings of expression difference of key biomarkers (Sox11 and Mlxipl) in PVM samples between *Sox11^+/+^* and *Sox11^+/-^* groups at protein expression level, we performed validation IHC experiments. The IHC experimental process of immunostaining had been described in previous studies[6 7]. Briefly, all paraffin-embedded PVM tissue samples from *Sox11^+/+^* and *Sox11^+/-^* mice were separated into two slices; one slice was immunostained with 1:50 diluted primary rabbit polyclonal anti-Sox11 diluent (Signalway AntiBody, College Park, USA) at 4 ℃ overnight, and the other one was immunostained with 1:100 diluted primary rabbit anti-Mlxipl diluent (Proteintech Group, Wuhan, China) overnight at 4 ℃. Then, after washing and staining in appropriate anti-rabbit secondary antibodies (DAKO, Carpinteria, CA) with diaminobenzidine substrate mixture (GeneTech, Shanghai, China) following the manufacturer’s instructions, PVM slices were visualized via an optical microscope equipped with a digital image capture system (Leica Camera, Wetzlar, Germany). Moreover, to determine the specificity of the primary Sox11/Mlxipl antibodies, PVM slides were incubated in negative control rabbit IgG without the primary Sox11/Mlxipl antibodies, and there was no non-specific immunostaining found in this condition. Finally, semi-quantitative analysis of the IHC-protein expression (IHC-P) score were performed by two independent experienced researchers with a blinded fashion using Image J software. In short, IHC-P score of each immunostained PVM slide were assigned scores separately by using Image J software and based on the stained area of IHC staining and intensity of IHC staining. Quantitation of the IHC staining extent of Sox11/Mlxipl was made as follows: 1, < 25% of the PVM tissue (sporadic); 2, 26%~50% of the PVM tissue (focal); 3, > 50% of the PVM tissue (diffuse). The IHC staining intensity of Sox11/Mlxipl was scored as follows: 1, weak staining (light yellow); 2, moderate staining (yellow brown); 3, strong staining (deep brown). Multiplication of the staining extent scores and staining intensity scores produces a semi-quantitative Sox11/Mlxipl IHC-P score for each PVM slice, with a minimum score of 1, and a maximum score of 9.

***In vitro* cell experiments**

To substantiate the *in vivo* evidence suggesting a role for Mlxipl overexpression in mitochondrial cristae disruption in PVM cells from Sox11^+/-^ mice, we constructed stable Mlxipl-overexpressing plasmids and generated corresponding stably transfected cell lines. SBI-piggyBac vectors and *Mlxipl* transient overexpression plasmids were purchased from Youming Biotechnology Co., Ltd, Guangzhou, China. To construct *Mlxipl* stable overexpression plasmids (SBI-*Mlxipl*), the complete coding sequence of mouse *Mlxipl* gene was subcloned into the SBI-piggyBac vectors. After transfecting C2C12 cell line, a common mouse myogenesis cell line *in vitro* widely used in many skeletal muscle studies[8 9], with constructed plasmids, the stably transfected cells were further screened via incubation with puromycin for 1 week. The stably transfected cell lines were named briefly as follows: OE-Mlxipl group (C2C12 transfected with SBI-*Mlxipl*) and Control group (C2C12 transfected with SBI-piggyBac empty vectors).

Then, the expression of Mlxipl in OE-Mlxipl and Control groups at mRNA and protein levels were examined via quantitative real-time reverse transcription polymerase chain reaction (qRT-PCR) and western blotting (WB), respectively.

**Quantitative real-time reverse transcription polymerase chain reaction (qRT-PCR)**

Briefly, for qRT-PCR, total RNA was extracted from C2C12 cells of OE-Mlxipl and Control groups via TRIzol reagent (Ambion, Austin, USA). The extracted RNA was transformed into cDNA and analyzed on ABI 7500 Real-Time PCR system (Applied Biosystems, Carlsbad, USA). The qRT-PCR primers used *in vivo* experiments were presented in **Supporting File 1**. Reactions were conducted in triplicate using SYBR Green Master Mix (TaKaRa, Osaka, Japan) and normalized with *Gapdh* mRNA level using ΔΔCt method.

**Western blotting (WB) analysis**

For WB, muscle cells were firstly sonicated with RIPA buffer (Beyotime Biotechnology, Shanghai, China) to harvest whole cell lysates. Rabbit polyclonal antibodies for Mlxipl were purchased from Proteintech Group, Inc (Wuhan, China), and for Gapdh were purchased from Signalway Antibody, Inc (College Park, USA). The expression levels of Mlxipl were quantified with Image J software and normalized to the Gapdh in the same sample.

Finally, we used TEM to observe the cellular changes among OE-Mlxipl, Control and Blank (C2C12 cells with PBS) groups. The cell samples process and data analysis were same as using TEM for PVM tissues observation mentioned above.

**Statistical analysis**

Results in current research were mainly shown as mean ± standard error (SE), except the results of longitudinal observations of cobb angle between different ages of *Sox11^+/+^* and *Sox11^+/-^* mice were expressed as mean ± standard deviation (SD). Statistical significance was all determined by the two-sided Student’s *t*-test. Statistical significance was defined as a P value < 0.05 across all analyses in this study.

**References**

1. Rong Z, Yang Z, Zhang C, et al. Bioinformatics analysis of paravertebral muscles atrophy in adult degenerative scoliosis. J Muscle Res Cell Motil 2023;**44**(4):287-97 doi: 10.1007/s10974-023-09650-8.

2. Dobin A, Davis CA, Schlesinger F, et al. STAR: ultrafast universal RNA-seq aligner. Bioinformatics 2013;**29**(1):15-21 doi: 10.1093/bioinformatics/bts635.

3. Liao Y, Smyth GK, Shi W. featureCounts: an efficient general purpose program for assigning sequence reads to genomic features. Bioinformatics 2014;**30**(7):923-30 doi: 10.1093/bioinformatics/btt656.

4. Love MI, Huber W, Anders S. Moderated estimation of fold change and dispersion for RNA-seq data with DESeq2. Genome Biol 2014;**15**(12):550 doi: 10.1186/s13059-014-0550-8.

5. Yu G, Wang LG, Han Y, He QY. clusterProfiler: an R package for comparing biological themes among gene clusters. Omics 2012;**16**(5):284-7 doi: 10.1089/omi.2011.0118.

6. Wu R, Tang W, Qiu K, et al. An Integrative Pan-Cancer Analysis of the Prognostic and Immunological Role of Casein Kinase 2 Alpha Protein 1 (CSNK2A1) in Human Cancers: A Study Based on Bioinformatics and Immunohistochemical Analysis. Int J Gen Med 2021;**14**:6215-32 doi: 10.2147/ijgm.S330500.

7. Tang W, Shao Q, He Z, Zhang X, Li X, Wu R. Clinical significance of nonerythrocytic spectrin Beta 1 (SPTBN1) in human kidney renal clear cell carcinoma and uveal melanoma: a study based on Pan-Cancer Analysis. BMC Cancer 2023;**23**(1):303 doi: 10.1186/s12885-023-10789-3.

8. Oprescu SN, Baumann N, Chen X, et al. Sox11 is enriched in myogenic progenitors but dispensable for development and regeneration of the skeletal muscle. Skelet Muscle 2023;**13**(1):15 doi: 10.1186/s13395-023-00324-0.

9. Shu Q, Yang G, Tang M, Guo C, Zhang H, Li J. Upregulated estrogen receptors impairs myogenesis and elevates adipogenesis related factor levels in the paravertebral muscles of patients with idiopathic scoliosis. Biochem Biophys Res Commun 2023;**652**:22-30 doi: 10.1016/j.bbrc.2023.02.034.
