# Supplementary material for: Sox11 deficiency induces paravertebral muscle injury and scoliosis via Mlxipl upregulation
Source: Genes Dis. 2025 Dec 12;13(3):101970. doi: 10.1016/j.gendis.2025.101970 (PMC12859181; doi:10.1016/j.gendis.2025.101970)
Supplement: Multimedia component 2 [file mmc2.docx]

**Figures legends for supplementary figures**

**Figure S1 *Sox11*^+/-^ mice show the short whole body with scoliosis phenotype since 12 week-of-age. (A)** After genotyping (**Supporting File 1,2**), the whole body length (74.67 ± 0.55 cm *vs* 77.28 ± 0.25 cm, *P* <0.01) of *Sox11*^+/+^ (WT) and *Sox11*^+/-^ (Het) mice were measured at 12 week-of-age. **(B)** Representative images of scoliosis phenotype (red arrow) in *Sox11*^+/-^ mice compared with *Sox11*^+/+^ mice at 12 weeks. **(C)** Representative lateral micro-CT images of *Sox11*^+/+^ and *Sox11*^+/-^ mice at 12 week-of-age. The presence of scoliosis at the level of thoracolumbar segment of spine was confirmed by micro-CT and indicated by red arrows. **, *P* <0.01; *ns*, *P* >0.05. Mirco-CT, microfauna computed tomography.

**Figure S2** **Normal vertebral bodies with reduced muscle volume of PVM in *Sox11*^+/-^ mice with scoliosis phenotype. (A)** Micro-CT analysis demonstrating 3D reconstruction of the spine in *Sox11*^+/+^ and *Sox11*^+/-^ mice. The vertebral bodies of *Sox11*^+/-^ mice exhibited no evidence of disruptive lesions or vertebral fusion abnormalities when compared to their *Sox11*^+/+^ littermates. **(B)** Spinal parameters, including spine length (39.83 ± 0.78 cm *vs* 38.57 ± 0.84 cm, *P* >0.05), spine volume (151.59 ± 4.62 mm^3^ *vs* 142.33 ± 6.45 mm^3^, *P* >0.05) and spine BMD (1.57 ± 0.02 g/mm^3^ *vs* 1.55 ± 0.01 g/mm^3^, *P* >0.05), were measured and compared between *Sox11*^+/+^ and *Sox11*^+/-^ mice. **(C)** The muscle volumes of PVM were measured by calculating green area and compared between *Sox11*^+/+^ and *Sox11*^+/-^ mice (162.95 ± 4.67 mm^3^ *vs* 185.86 ± 4.39 mm^3^, *P* <0.01). **, *P* <0.01; *ns*, *P* >0.05. Mirco-CT, microfauna computed tomography; BMD, bone mineral density; PVM, paravertebral muscle.

**Figure S3** **Overexpression of Mlxipl in developmental PVM is related to lipometabolism dysregulations and mitochondrial cristae malformations in *Sox11*^+/-^ mice.** GO enrichment analysis was conducted on downregulated (A) and upregulated (B) genes (**Supporting Files 3 and 4**) in developing PVM samples derived from littermate Sox11^+/+^ and Sox11^+/-^ mice. The top 10 statistically significant GO biological process terms are displayed to the left of each bubble plot. **(C)** Literature review analysis has elucidated potential associations between the functions of upregulated genes and the regulation of lipid metabolism as well as mitochondrial cristae biogenesis. Among the 15 analyzed upregulated genes, only the *Mlxipl* gene was highlighted (as indicated by the red box) to exert significant dual roles in both lipid metabolic regulation and mitochondrial cristae formation. **(D)** Quantity evaluation of Sox11 (2.33 ± 0.33 *vs* 7.50 ± 0.67, *P* <0.001) IHC-P value in PVM slices of *Sox11*^+/+^ and *Sox11*^+/-^ mice. *, *P* <0.05; ***, *P* <0.001. GO, gene ontology; PVM, paravertebral muscle; IHC, immunohistochemistry; IHC-P, immunohistochemistry protein expression.

**Figure S4 WB analysis further confirmed the overexpression of Mlxipl in transfected OE-Mlxipl cells *in vitro*.** The representative uncropped WB gel images with molecular weight markers for **(A)** Mlxipl and **(B)** Gapdh.
